# Anomalous Clouding Behavior of Polysorbate 80—Deciphering the Role of Nonesterified Components

**DOI:** 10.1021/acs.molpharmaceut.4c01268

**Published:** 2025-05-14

**Authors:** Alaa Hassan, Tim Diederichs, Patrick Garidel, Heiko Heerklotz

**Affiliations:** † Institute of Pharmaceutical Sciences, Department of Pharmaceutics, University of Freiburg, Hermann-Herder-Str. 9, Freiburg 79104, Germany; ‡ PDB-TIP, Innovation Unit, Boehringer Ingelheim Pharma GmbH & Co. KG, Birkendorfer St. 65, Biberach an der Riss 88397, Germany; § Martin Luther University Halle-Wittenberg | MLU · Institute of Chemistry, Physical Chemistry, von-Danckelmann-Platz 4, Halle D-06120, Germany; ∥ Leslie Dan Faculty of Pharmacy, University of Toronto, 144 College Street, Toronto M5S 3M2, Ontario, Canada; ⊥ Faculty of Pharmacy, Cairo University, Kasr El-Aini St., Cairo 11562, Egypt

**Keywords:** Tween 80, LCST, cloud point, dewetting, surfactant-rich phase, pseudoternary phase diagram, PEG 400, Renex S30

## Abstract

Polysorbates (PSs) are key excipients for the colloidal stability of biopharmaceuticals with unique properties. A comprehensive understanding of the physicochemical properties of these multicomponent products is essential to address potential stability issues without compromising their functionality. Here, we demonstrate that polysorbate 80 HP (PS80) shows an anomalous clouding, i.e., a thermotropic liquid–liquid phase separation behavior, which cannot adequately be explained by the conventional interpretation assuming a pseudobinary system. In a binary two-phase system of surfactant and buffer, an increase in the total surfactant concentration increases the fraction of the surfactant-rich phase in the respective proportion (lever rule). PS80 within about 7 K of the lower critical solution temperature fails to comply with this; concentrations and compositions of the coexisting phases change with the total concentration. This renders the phases more alike and, at some point, eliminates phase separation. This significant deviation from the pseudobinary phase behavior can be resolved by conceptually dividing the numerous chemical species in PS80 into two independent pseudocomponents, PS80-I and -II. Ternary phase diagrams derived from this approach successfully explain the observed anomalous behavior. RP–UPLC–MS analysis indicated a concentration-dependent redistribution of the nonesterified components (NECs), suggesting, along with other evidence, that NECs are key constituents of component II. Specifically, free polyethylene glycol (PEG) and/or PEG-sorbitans seem to function as intrinsic cosurfactant(s) within PS80, modulating its wetting and clouding properties. The latter is important for interaction, association, and phase separation properties in biologics.

## Graphical abstract

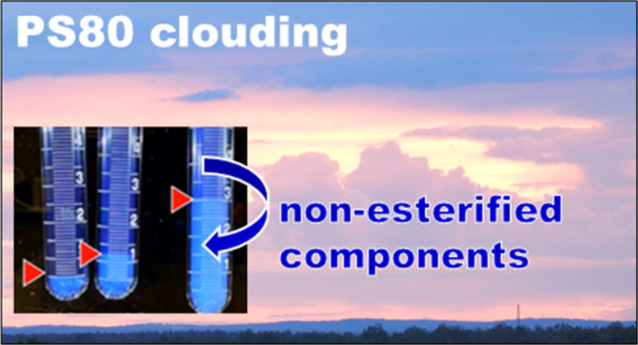

## 1 Introduction

The development of biopharmaceuticals is challenged by the need to stabilize the drug product with proper excipients while also ensuring the stability and safety of these excipients. Virtually, all marketed parenteral biotherapeutics contain nonionic surfactants^1^ such as polysorbates 20 (PS20) and 80 (PS80) and poloxamer 188.^2–4^ Their key function is to cover product–air and product–container interfaces, thereby preventing protein particle formation and denaturation at these interfaces.^5–8^ In line with these effects, PSs stabilize biologics upon agitation,^9–11^ freezing and thawing,^12^ and lyophilization.^13,14^ Polysorbate concentrations in the range of 0.1–1 mg/mL^15,16^ or even as little as 0.01–1 mg/mL^17^ were found sufficient for this purpose. Commercially available compendial PSs primarily consist of polyoxyethylene-1,4-sorbitan monoesters of fatty acids (as illustrated in Figure 1). However, they also include various other combinations of their constituent building blocks, including isosorbide compounds, unesterified POE-sorbitans, free polyethylene glycols, fatty acids, and tri- and tetra-esters^18–20^ (for more details, see Figure S-1 in Supporting Information). The predominant fatty acid in all PS80 products is oleic acid. PS80 comes in various purity or composition standards, including “high-purity” (HP), “China-grade”, “super-refined”, or “pure oleic acid”. Here, we focus on the compendial HP standard (see Table S-1 for the fatty acid distribution in PS80 HP) and refer to it as “PS80”.

**Figure 1:**
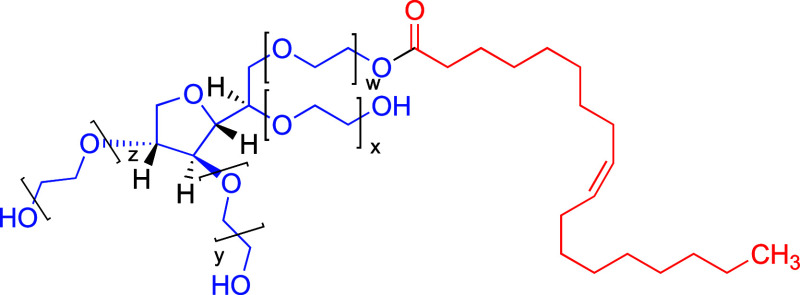
Idealized structure of polysorbate 80 (PS80). The blue section represents the hydrophilic ethoxylated sorbitan ring, where *w* + *x* + *y* + *z* = 20 is the average total number of EO units; the red section represents the hydrophobic main fatty acid ester tail (oleic acid) (sketched with ChemDraw).

Generally, PSs have a good safety profile.^21–23^ However, hydrolysis and oxidation of the fatty acid esters can result in different levels of impurities and degradation products. This structural heterogeneity necessitates thorough scrutiny to ensure uniform behavior within a given grade of a given PS.^24^ In recent years, issues relating to the oxidative and enzymatic degradation of polysorbates have been raised, with implications for the stabilization of biologics.^25,26^ Either improvement or replacement of PS in a given formulation requires a profound understanding of the unique properties of these compounds.

The heterogeneous nature of PS80 presents a technical challenge, yet it also renders PS80 a product with unique and, in some respects, superior properties that fundamentally deviate from the standard behavior of “a surfactant”. The inability of most surfactant textbook knowledge to adequately explain the physicochemical characteristics of PS80 complicates the understanding of its performance and potential issues. For example, we have demonstrated recently that it is pointless to deal with a CMC for PS80.^27^ Throughout and beyond the practically relevant concentration range between 10 μM and 10 mM, PS80 in buffer resembles neither a “surfactant solution below the CMC” nor a “surfactant dispersion above the CMC”. That is, there is neither a solution of surfactant monomers without micelles nor a constant monomer concentration in equilibrium with micelles of characteristic size and shape. Instead, PS80 undergoes a continuous association process over many orders of magnitude in concentration, with each of its various chemically distinct species entering micelles in a specific concentration range.^27^

The aim of this study is to report and interpret another anomaly of PS80 that impacts its wetting and clouding behavior. Clouding (CL) refers to the spontaneous phase separation of a surfactant solution as it reaches a characteristic, concentration-dependent temperature, *T*_CL_. Above this temperature, the sample is composed of a surfactant-rich phase (SRP) and a surfactant-poor phase (SPP). The minimum of *T*_CL_ is denoted as the lower critical solution temperature (LCST), while *T*_CL_ at 1% surfactant is defined as the cloud point. Upon two-phase coexistence involving two components, increasing the total surfactant concentration must favor the SRP over the SPP at the respective proportion. In contrast to this, we will show below that in the case of PS80 right above the clouding temperature, the concentration of the SRP drops with the increasing total surfactant concentration so that the formation of SRP proceeds in a nonlinear fashion and phase separation vanishes well below the originally projected concentration. This indicates that, in terms of the clouding behavior, PS80 cannot be treated like a single-component surfactant or even a family of closely related, similar surfactants forming a single “pseudo-component.”

It is important to note that clouding can have several potentially significant direct and indirect consequences for pharmaceutical applications. Phase separation can sort and concentrate active ingredients, alter their surface properties, and potentially promote aggregation.^28–31^ The clouding behavior of PS80 itself can pose issues, such as during the autoclaving of formulation components. Additionally, since the clouding temperature can be significantly lowered by certain additives,^32^ it may even affect the temperature range within which the final protein-containing product is to be handled. Temperature- and composition-driven dewetting effects may also alter the surface coverage, surfactant–protein interactions, and micellar topology,^33^ which may strongly affect viscosity. Finally, the liquid–liquid phase separation (LLPS)^28–30^ of therapeutic proteins is a distinct phenomenon but likely related to clouding by involving the dewetting phenomena of excipients.^31^

Given our aim to understand anomalies in the clouding behavior of PS80 that are of relevance for liquid pharmaceutical dispersions, primarily biologics, we will focus on the thermodynamics of aqueous dispersions at a high water content. How the molecular phenomena presented here might affect the structure and phase behavior of low-water PS80 systems with some potential interest for other dosage forms may be a topic for follow-up studies with a different aim and approach.

Various methods have been employed to study clouding-related properties, including light scattering,^34,35^ refractometry,^36^ turbidimetry,^37^ viscometry,^38^ thermo-optical methods,^39^ and visual inspection.^40,41^ However, a challenge with some of these methods is their inability to clearly distinguish between different dewetting-related but principally distinct phenomena, such as micellar growth including thermotropic sphere-to-rod transitions,^42^ critical fluctuations, and a true macroscopic phase separation. Given our focus on the latter, we chose visual inspection as the most reliable method. Additionally, changes in the composition of the phases were monitored using reverse-phase ultra-high-performance liquid chromatography coupled with mass spectrometry, RP–UPLC–MS.

## 2 Materials and Methods

### 2.1 Materials

Polysorbate 80 of compendial high-purity grade (PS80 HP) was obtained from Croda Health Care (Edison, NJ, USA), which complies with Ph. Eur., USP/NF, and JP standards.^43^ A 25 mM citrate buffer (pH, 6.0) containing 115 mM NaCl was used in line with parenteral formulations. Reagents of analytical grade were sourced from Merck KGaA (Darmstadt, Germany), and ultrapure water (18.2 MΩ cm resistivity) was used for the buffer solution. BioUltra polyethylene glycol 400 (Merck KGaA, Darmstadt, Germany) and Renex S30-LQ-(MV) ETR2030/SAMP (Croda, Spain) were purchased to perform subsequent confirmatory visual inspection tests.

Typically, a stock solution of 210 mg/mL was prepared by topping up an appropriate mass of PS80 with the buffer used to a total volume of the stock solution. For handling and comparison purposes, the corresponding primary concentration measure in mg/mL solution is then converted into molarity, *c*_PS80_ ≈ 160 mM, using an effective molar mass of PS80 HP of 1310 g/mol, and into mass fraction, *X*_PS80_ ≈ 20.8%, according to the density of PS80 dispersions in citrate buffer at room temperature (25 °C) of ≈1.01 kg/L. That means, concentrations given in % always refer to mass percent. Densitometric measurements of PS80 HP at concentrations of 10–100 mM in citrate buffer at 25 °C yielded a partial specific volume of 895–900 mL/g of PS80,^44^ which converts into a total density of 998–1012 g/L for the whole dispersions and ≈1020 g/L for 160 mM PS80.

### 2.2 Visual Inspection

A series of dilutions of the stock solution with buffer were prepared in 10 mL Pyrex tubes (NS 12.5/21, Nr.42766010) and sealed with stoppers and parafilm (M Laboratory Film, Pechiney Plastic Packaging, Chicago, USA) to prevent evaporation. To minimize oxidative degradation, the samples were kept under Argon gas (5.0 purity) from Sauerstoffwerk (Friedrichshafen, Germany).

The dilutions were subjected to a controlled temperature program, incrementally increasing from 70 to 95 °C typically in 1 K steps. A thermostatic water bath (Erweka GmbH, Type: DT) with a custom cover was used to equilibrate the samples at each given temperature. After 4 h of incubation, the presence and volume fraction of the SRP (φ_SRP_) was inspected. Temperature homogeneity across the water bath was ensured by monitoring with a Testo 720 thermometer at multiple positions. Upon setting a new incubation temperature, samples were homogenized again by inversion to ensure an active phase separation at each given temperature.

### 2.3 Reversed-Phase Ultra-High-Performance Liquid Chromatography–Mass Spectrometry

Different concentrations of PS80 HP were prepared in citrate buffer, and reference samples were collected at room temperature. Then, 10 mL of each sample was incubated at 85 °C. After a 4 h incubation, SPP and SRP samples were carefully collected using 1 mL Omnifix syringes (B. Braun Melsungen, Germany) to avoid disturbing the phase separation. Each sample was diluted in a defined fashion to match the suitable range for the measurement, typically in the range of 0.05–0.6 mg/mL.^45,46^

All collected samples were analyzed using the reverse-phase ultraperformance liquid chromatography–mass spectrometry (RP–UPLC–MS) method adapted from Lippold et al. (2017)^45^ and Evers et al. (2020).^46^ The analysis was conducted on an Ultimate 3000 UHPLC system (Thermo Fisher Scientific, Waltham, MA, USA), equipped with an RS dual gradient pump, an RS autosampler, and an RS column compartment coupled with an ACQUITY QDa mass detector (Waters Corporation, Milford, MA, USA) featuring an electrospray ionization (ESI) source. Polysorbate subspecies were separated using a Poroshell 120 SB-C8 4.6 × 100 mm, 2.7 μm reversed-phase column (Agilent Technologies, Inc., Santa Clara, CA, USA) with three mobile phases (A: 100% acetonitrile, B: 100% ultrapure water, and C: 100% methanol) at a flow rate of 0.7 mL/min. Postseparation, the analytical gradient was mixed with a 10 mM ammonium formate buffer, delivered by a second pump at 0.2 mL/min using a T-piece. A 10 μL sample injection was used, and the column oven was maintained at 60 °C with a total run time of 45 min. The QDa detector performed a mass scan from 250 to 1250 Da in positive mode at a sampling rate of 2 Hz. Measurements were calibrated against PS80 standards ranging from 0.05 to 0.6 mg/mL, with a limit of quantification of 0.05 mg/mL.

That means, MS was used for two purposes. First and routinely, the added mass of all components eluting at a certain elution time was determined by MS; the intensity representing the ordinate of Figure 4A corresponds to the cumulative intensity of mass scans ranging from 250 to 1250 Da for the SRP. Second, MS was used to identify the individual chemical species eluting at a given time, i.e., to assign groups of compounds to the chromatographic bands. To this end, the RP–UPLC–MS peak assignment of whole PS80 ^45,47^ was repeated for the individual phases appearing above the clouding temperature.

## 3 Results

### 3.1 Visual Inspection at ≥ 80 °C

At 85 °C, all samples between 2.5 and 210 mM PS80 exhibited separation into a bluish opalescent phase at the bottom and a clear one on top (Figure 2A). The concentration trend implies that the bottom phase is the SRP; its volume fraction, φ_SRP_, increases with the total concentration of PS80. Figure 2B shows data from five sets of samples. Values of φ_SRP_ below 5% as obtained for 2.5 and 5 mM (left two vials in Figure 2A) were too small to be precisely quantified in our setup.

**Figure 2:**
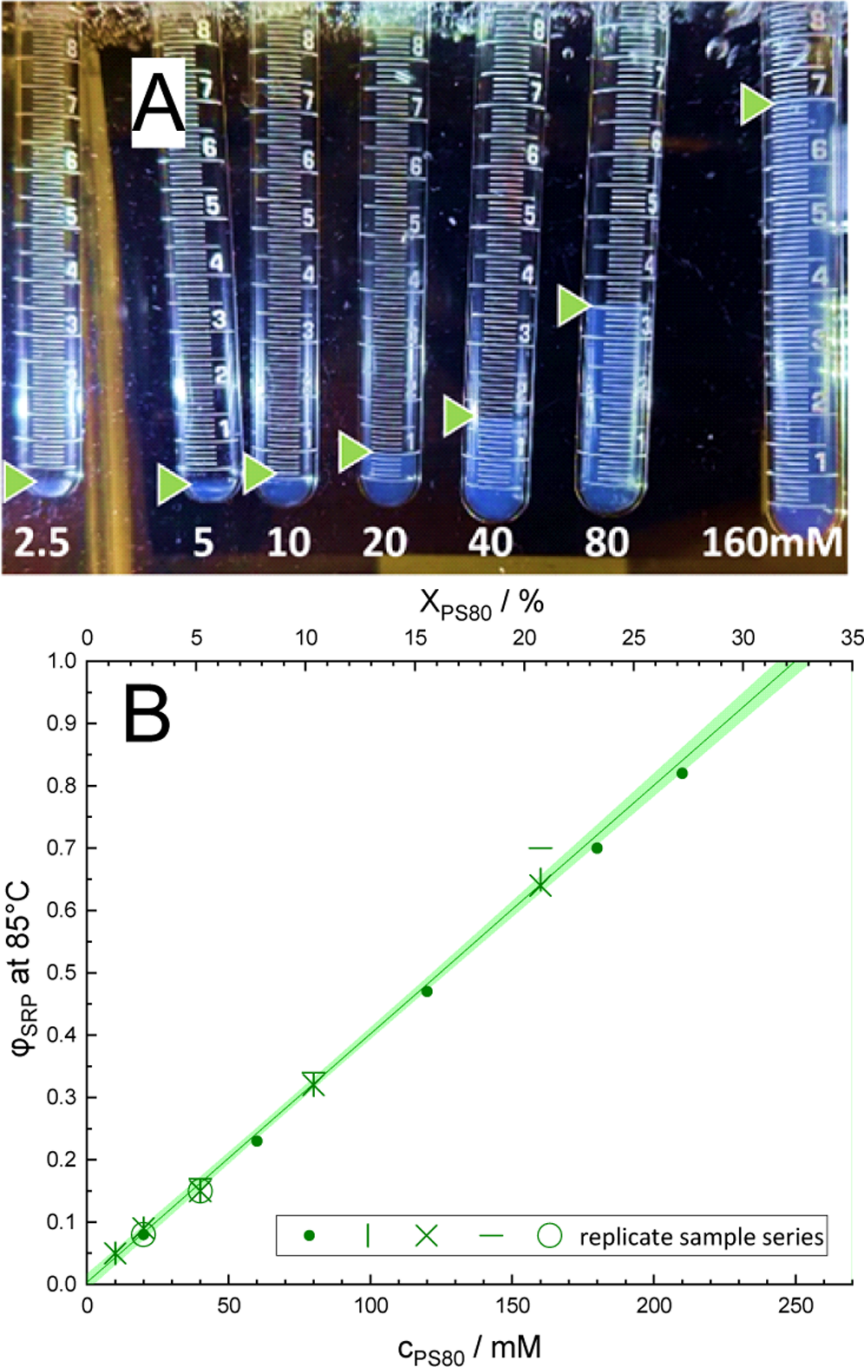
Clouding behavior of PS80 at 85 °C. (A) Set of serial dilutions (2.5, 5, 10, 20, 40, 80, and 160 mM) of PS80 HP in 25 mM citrate buffer, pH 6, equilibrated in a water bath at 85 °C. Note the increasing volume fraction of the SRP, φ_SRP_, with concentration. The results are included in B as horizontal bars. (B) φ_SRP_ read from the samples in panel A (horizontal bars) and four additional, independent sets of samples (other symbols) along with a linear fit and 95% confidence interval for all data points. The end points of the regression line, at φ_SRP_→0 and φ_SRP_→1, are shown in Figure 6 and Figure S-4 as open and solid black spheres, respectively. Other examples showing analogous pictures to 2A for 81, 83, and 87 °C are collected in Figure S-2.

A linear regression analysis of the data presented in Figure 2B was conducted to predict *c*_PS80_ in the SPP and SRP at 85 °C. This prediction can be achieved in a pseudobinary system by linearly extrapolating to φ_SRP_ = 0%, which corresponds to the inferred *c*_PS80_ in the SPP at 85 °C, reached in a 95% confidence interval of 0–2.4 mM (*X*_PS80_ ≤ 0.4%), and to φ_SRP_ = 100%, which corresponds to the predicted *c*_PS80_ in the SRP at 85 °C, reached at 250 ± 7 mM, i.e., 32.3 ± 0.8%. These values represent the concentration of PS80 at the boundaries of the two-phase range at 85 °C. By applying the same methodology to the studied temperature range (see Figures S-2 and S-3), we were able to set the boundaries displayed in the pseudobinary phase diagram as black spheres (open black spheres: SPP, solid black spheres: SRP); see Figure 6 and Figure S-4 for details.

### 3.2 Visual Inspection Closer to the LCST—Anomalous Clouding Behavior

A qualitatively different observation was made closer to the LCST in the temperature range of 70 °C < *T* < 77 °C. As depicted in Figure 3 for 75 °C, from 2.5 to 40 mM PS80, a proportional increase in φ_SRP_ is found, resembling the behavior found at 85 °C (Figure 2). It extrapolates to an apparent 95% confidence interval of 117 ± 8 mM at φ_SRP_→1 (*X*_PS80_(SRP) = 15 ± 1%). However, in stark contrast to the higher temperature findings and expectations from the lever rule, the 80 mM sample is turbid throughout, as one would expect close to a critical point. The 160 mM sample is completely clear. Figure 3B compiles results from several sets of fresh samples, including those at intermediate concentrations. It reveals a highly nonlinear behavior, suggesting a decreasing concentration of the SPR and, as phases get more similar, a nonlinear progress of SRP formation. This contrasts with the pseudobinary behavior of PS80 buffer observed at 85 °C (Figure 2).

**Figure 3:**
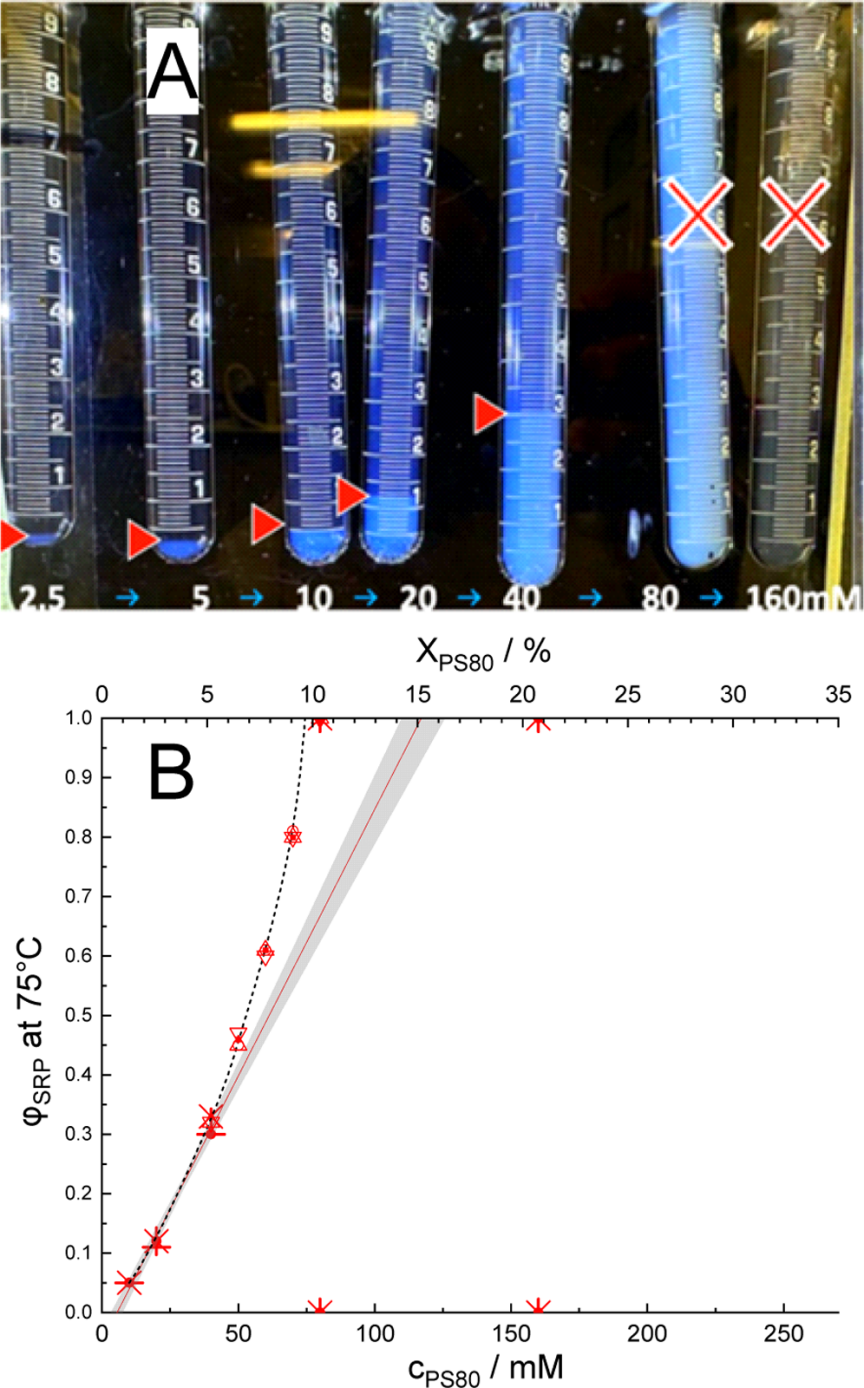
Clouding behavior of PS80 at 75 °C. (A) Set of serial dilutions of PS80 in 25 mM citrate buffer, pH 6, equilibrated at 75 °C, with increasing fractions of SRP up to 40 mM. Note the lack of phase separation at higher concentrations, contrasting with the behavior above 80 °C. (B) Volume fraction of the SRP, φ_SRP_, obtained from the samples in panel A and six additional, independent batches (different symbols). A linear fit of all data up to 40 mM and 95% confidence interval is shown in red and gray. The end points of the regression line, at φ_SRP_→0 and φ_SRP_→1, are shown in Figures 6 and S-4 as open and solid black spheres, respectively. The arbitrary short-dashed line illustrates the nonlinear behavior of the system (to guide the eye only). Examples for analogous pictures to 3A at 71, 73, and 77 °C are collected in Figure S-2.

### 3.3 Characterization of the Compositions of the Separated Phases by RP–UPLC–MS

We utilized reverse-phase liquid chromatography coupled to mass spectrometry (QDa) to screen the compositions of both separated phases (SPP and SRP samples), as well as nonseparated samples used as references.

For elution times of 16 min and more, when the esterified components (ECs) of PS80 elute, the positions and relative proportions of the bands in the elution profile, as well as the MS-based assignment of the individual bands to groups of compounds (sharing fatty acid(s) but varying in EO numbers), were in line with the published data for the overall PS80 HP product. Refer to refs 45 and 47 for a more detailed peak assignment of the chromatograms. Most important for the study presented here is that chromatograms of the SRP collected at different total concentrations of PS80 indicate a pronounced relative change of the earlier-eluting, more polar, nonesterified components (NECs) compared to other peaks arising from the ECs of PS80 (Figure 4A). These NECs consist of free low-molecular-weight polyethylene glycol chains (LMW-PEG) and nonesterified polyols (POE-sorbitan and POE-isosorbide)^45,48^ (representing species 1 in the study of Sun et al.;^49^ see Figure S-1). The complex elution profile between 18 and 27 min (Figure 4A) represents the wide variety of ECs within PS80 (from species 2 to 9 in the study of Sun et al.;^49^ see Figure S-1). There is minimal variation in the relative content of EC species within the SRP, possibly because, already at the lower concentrations tested, nearly all EC molecules reside in the SRP.

**Figure 4:**
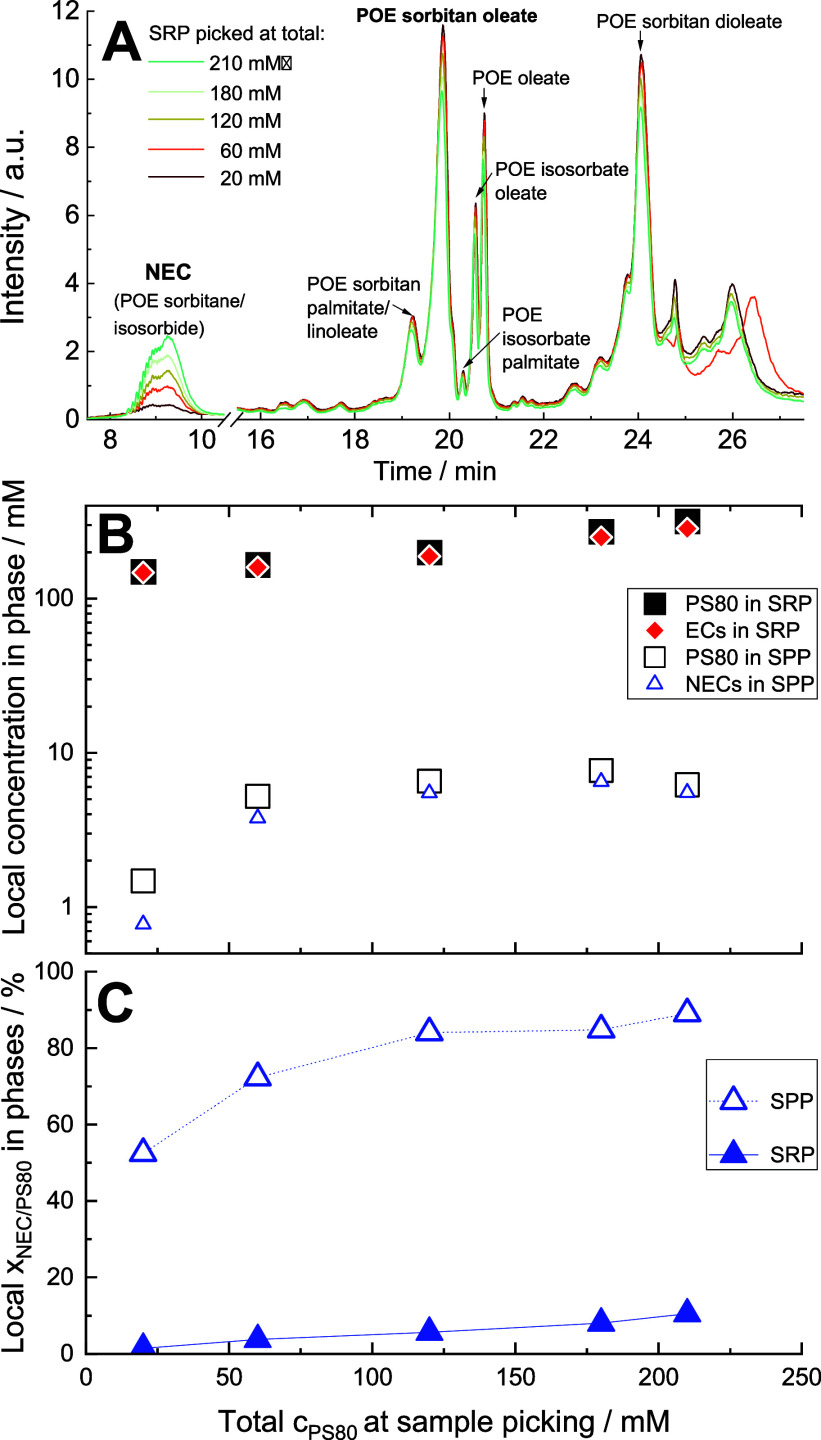
Results of RP–UPLC–MS experiments, given as a function of the total PS80 concentration, *c*_PS80_, of the samples. (A) Chromatograms (normalized with respect to concentration) of SRP samples. Note the consistent change of the broad, first eluting peak left of the axis break, which represents a variety of nonesterified compounds (NECs), with increasing the total concentration of PS80. Some assignments of bands are provided; for more detail, see refs 45, 47. (B) Local concentration of all PS80 in SRP (solid squares) and SPP (open squares); solid diamonds for the esterified compound (EC) concentration in SRP and open triangles for NECs in SPP indicate that these are the major fractions of PS80 in the respective phases. (C) Mass fractions of NECs relative to total PS80 in the SRP and SPP. Symbols are defined in the plot windows.

The total concentrations of PS80 (ECs + NECs) in the SPP and SRP collected at 85 °C are shown as black open and solid squares, respectively, in Figure 4B. With respect to their order of magnitude, these concentrations are in line with the predictions extrapolated from Figure 2B and analogously at other temperatures (Figure S-3). The SPP is quite dilute, with most of its PS80 being NECs (depicted as open blue triangles in Figure 4B). Conversely, the SRP, ranging from 150 to 300 mM, mainly consists of ECs (shown as solid red diamonds in Figure 4B). It is important to note that, unlike a two-component system, these concentrations vary considerably with the increasing total concentration of PS80.

The relative changes in the composition of PS80 in the coexisting phases are more clearly illustrated in Figure 4C, which shows the mass fraction of NEC relative to total PS80 (denoted by *x*) in both phases. Self-association studies have shown that as the concentration of a PS80 dispersion increases, more PS80 species are attracted to enter micelles in the order of increasing individual CMC (decreasing lipophilicity).^27^ Therefore, it is plausible that with the increasing PS80 concentration, the few remaining ECs in SPP tend to redistribute in favor of the SRP, resulting in an increased fraction of NECs in the SPP (open blue triangles in Figure 4C). The same effect may also explain the observation that the originally all-polar NECs, which tend to dewet with the increasing temperature as well,^50^ are also gradually incorporated into the SRP, thereby increasing its local content (solid blue triangles in Figure 4C).

The overall content of NECs in PS80 HP, as determined from the reference samples collected without phase separation, amounts to 12% of the total mass. This aligns with the standard NEC content of PS80 HP.^49^

### 3.4 Effect of Adding Free NEC Compounds on the Clouding of PS80

Subsequent visual inspection after the external addition of free NEC subspecies confirmed that the NEC content has a strong effect on the anomaly in the clouding behavior of PS80. Two representative candidates were investigated: PEG 400, representing free low-molecular-weight polyethylene glycol (LMW-PEG, Figure 5A), and sorbeth 30 (marketed as Renex S30, Figure 5B) as a representative for PEG-sorbitanes.

**Figure 5:**
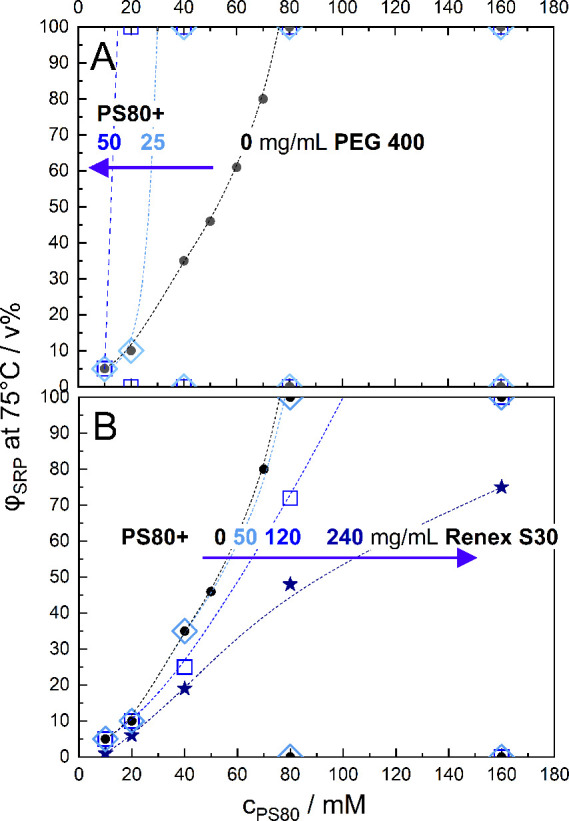
Influence of adding the nonesterified compounds (NECs) PEG 400 and Renex S30 on the clouding behavior of PS80 at 75 °C. The volume fraction of the SRP, φ_SRP_, is plotted as a function of the molarity of PS80, *c*_PS80_. Black spheres in both panels reproduce the result for PS80 alone from Figure 3A. (A) The presence of additional PEG-400 at 25 (light blue diamonds) and 50 mg/mL (blue squares) renders the progress of PS80-concentration-driven SRP formation steeper and nonlinearly enhanced at high PS80. 100 mg/mL PEG 400 eliminates all visible phase separation in the range investigated (not shown explicitly). (B) The addition of Renex S30 at 50 (light blue diamonds), 120 (blue squares), and 240 (navy stars) mg/mL renders the PS80-induced transition less steep, i.e., increases the PS80 concentration in the SRP, and changes the principal nonlinearity.

In Figure 5A,B, the solid black spheres connected by an arbitrary dashed line to guide the eye (with the constraint to reach φ_SRP_: = 1 between 70 and 80 mM) reproduce the data for PS80 from Figure 3B. The addition of PEG 400 (Figure 5A) at all tested concentrations consistently renders the progress of φ_SRP_(*c*_PS80_) steeper, i.e., additional PEG 400 causes the PS80 concentrations of the two phases to approach each other. At 100 mg/mL PEG 400, phase separation is entirely inhibited across all of the investigated PS80 concentrations.

Addition of Renex S30 (Figure 5B) has the opposite effect. The slope of φ_SRP_(*c*_PS80_) gets shallower and, at 120 mg/mL Renex, closer to linear than that in the absence of extra Renex S30. At 240 mg/mL, the shape of the curve changed to a decreasing slope. That means, additional Renex S30 renders the two phases more different in *c*_PS80_ and, hence, broadens the two-phase range. Note that the addition of Renex S30 also reduces the density of the SRP relative to the SPP, making it float to the top when 240 mg/mL Renex S30 were added.

Summarizing, NEC compounds have a strong effect on PS80 clouding by rendering the properties of the coexisting phases more alike (PEG 400) or more different (Renex S30). Hence, the redistribution of the intrinsic NEC components of PS80 between the phases with increasing *c*_PS80_ must be considered to affect clouding as well.

## 4 Discussion

### 4.1 Limited Applicability of the Pseudobinary Phase Diagram of PS80 and Buffer

Binary or pseudobinary phase diagrams representing thermotropic liquid–liquid phase separation have been published for many pure surfactants and technical surfactant products, which are mixtures of related compounds (see Figure S-5 for a compilation of phase diagrams of PONPE 7.5,^51^ TX-114,^52^ TX100,^53^ and Poloxamine 908 ^54^). Since phase separation cannot occur over extended temperature ranges for the pure phases (*X* = 0 and *X* = 1) and is most favorable at a certain composition, *X*_LCST_, the composition-dependent phase separation (clouding) temperature, *T*_CL_(X), typically decreases to a minimum referred to as the LCST and then increases again. Depending on whether *X*_LCST_ of a given surfactant is below, within, or above the concentration range studied, the phase diagrams show the increasing or decreasing branch of the clouding boundary, *T*_CL_(*X*_PS80_), or both. We attempted to determine the phase boundaries in a putative, pseudobinary phase diagram of PS80 and citrate buffer by two methods referred to as “vertical” and “horizontal”.

The “vertical” approach recorded the temperature at which the first appearance of a separate SRP could be detected for a series of samples with different *c*_PS80_. These *T*_CL_ values are depicted in Figure 6 as black triangles. Apparently, *X*_LCST_ of PS80 is below the concentration range investigated so that the figure shows the increasing branch of *T*_CL_. The second, “horizontal” approach makes use of the lever rule for a two-phase region of a pseudobinary system. It requires, at a fixed temperature, that the fraction of the higher concentrated phase, here, φ_SRP_, must vary linearly with the total concentration, *X*_PS80_, ranging from 0 at the left (lower-*X* boundary) to 1 at the right (higher-*X* boundary). Thus, the boundaries at a given temperature can be obtained by a linear extrapolation of *X*_PS80_(φ_SRP_) to φ_SRP_→0 to yield *X*_PS80_ in SPP and φ_SRP_→1 to yield *X*_PS80_ in SRP, as demonstrated in Figures 2B, 3B, and S-3. The results are depicted in Figure 6 as black open spheres for the SPP and solid spheres for the SRP; a reproduction of the figure with a logarithmic abscissa to better resolve the low-concentration range is given in Figure S-4.

**Figure 6:**
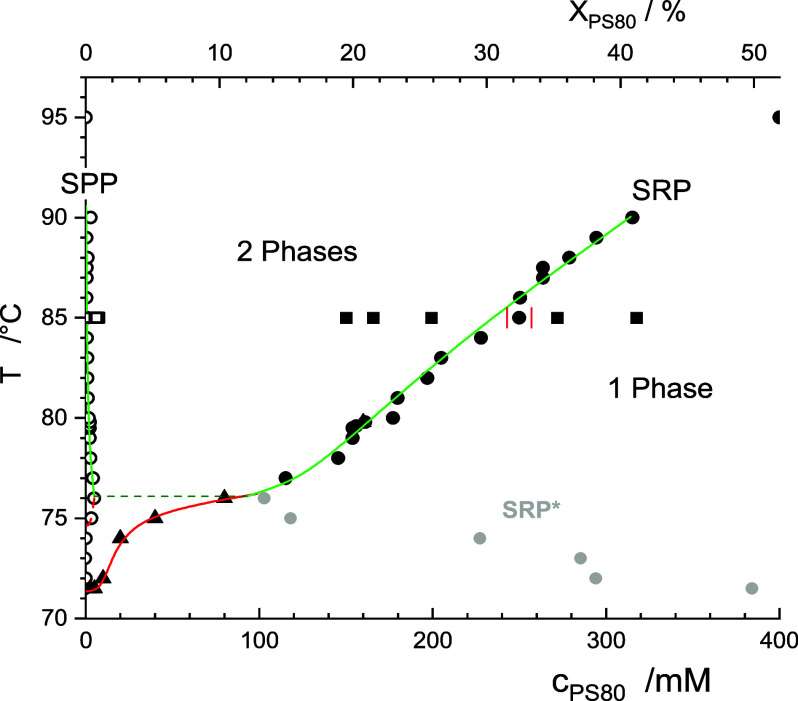
Pseudobinary phase diagram of PS80 and 25 mM citrate buffer including 115 mM NaCl, pH 6, showing the onset temperature of visible phase separation (black triangles) and prorated concentrations of the surfactant-poor phase (SPP, open black circles) and surfactant-rich phase (SRP, solid black spheres) enclosing the two-phase range. Gray spheres denoted SRP* represent apparent SRP concentrations estimated at a low concentration but lying within a single-phase range. Black squares indicate results from UPLC–MS converted from the data in Figure 4. Axes represent the temperature, *T*, and the concentration of PS80 given as molarity, *c*_PS80_ (bottom), and mass fraction, *X*_PS80_ (top). Lines are to guide the eye only.

As two principally equivalent approaches to determine the phase boundaries in a pseudobinary system, the black solid triangles (obtained by the vertical approach) and solid sphere (obtained by the horizontal approach) should ideally align along common, smooth lines (Figure 6). This alignment holds true above 80 °C but clearly not up to 76 °C, where the extrapolated concentrations of PS80 in the SRP are in a range up to 400 mM (labeled SRP* in Figures 6 and S-4), yet there is no phase separation at those concentrations. This is an unequivocal demonstration that at least up to 76 °C, PS80—citrate buffer cannot, even approximately, be treated as a pseudobinary mixture.

These findings suggest the possible presence of a third independent pseudocomponent within the PS80–citrate system, which would render the composition of the SRP at a fixed temperature dependent on concentration. The RP–UPLC–MS data at 85 °C, along with the subsequent confirmatory visual inspection of the effect of adding free NEC compounds on the clouding of PS80, indicated that this third pseudocomponent is likely governed by NEC or compounds of the NEC family (see blue triangles in Figure 4C). This raises the question of whether similar anomalies could be represented in the phase diagram of other surfactants, such as TX-114, which also exhibits a steep phase boundary before the ascending branch of its binary phase diagram^52^ (see Figure S-5). It should be noted that the inconsistency of the pseudobinary phase diagram can only be detected by the horizontal approach, not the vertical approach mentioned earlier.

### 4.2 PS80–Citrate Buffer as a Pseudoternary System

The failure of the pseudobinary phase diagram suggests the need to consider at least one additional, independent pseudocomponent in PS80. Given the extreme chemical diversity within PS80, certain ingredients cannot be lumped into a single pseudocomponent. Thus, we propose dividing PS80 into two distinct pseudocomponents, PS80-I and PS80-II. The limitations of the pseudobinary approach likely arise from a concentration-dependent redistribution of these pseudocomponents between phases, as observed in Figure 4C for NECs. Based on the RP–UPLC–MS data at 85 °C, PS80-II is tentatively identified with NECs or related compounds, while PS80-I could be associated with the grouped EC components.

To demonstrate the role of pseudocomponent II, we constructed pseudoternary phase diagrams of PS80-I, PS80-II, and buffer solution that would account for the anomalous and more typical clouding behavior of PS80 seen at 75 and 85 °C, respectively. Mass fractions referring to the total system, *X*_PS80‑I_, *X*_PS80‑II_, and *X*_buffer_, were estimated based on a density of ≈1 kg/L for all phases and setting the mass fraction of II within PS80 to *x*_II_ = *X*_PS80‑II_/(*X*_PS80‑I_ + *X*_PS80‑II_): = 12%. This percentage is that of NEC within PS80 according to the literature^49^ and the UPLC-MS data of this study. If, for example, only part of the NECs contributed to II, this would change the absolute values in the phase diagram but not the principal behavior to be discussed here. What is primarily important is that the proportion of I and II is fixed in all of our PS80-only samples, so that all samples represent points (blue spheres) on a straight line (dashed blue in Figure 7) starting at the buffer corner, where *X*_PS80‑I_ = *X*_PS80‑II_ = 0.

**Figure 7:**
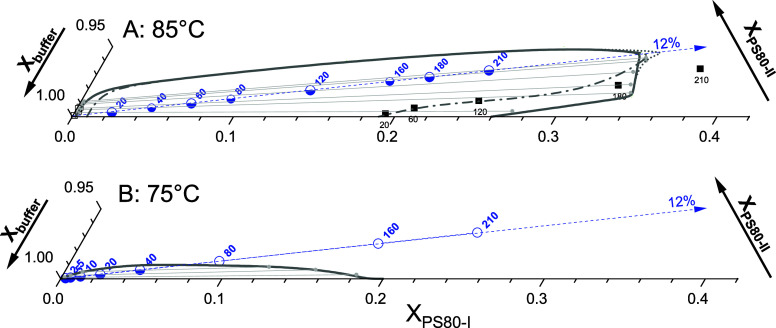
Semi-schematic ternary phase diagrams compatible with the findings of visual inspection at 85 °C (A) and 75 °C (B). The diagrams illustrate the phase behavior of the system at the specified temperatures. Blue spheres represent the total compositions of the samples, under the assumption that PS80 needs to be divided into two pseudocomponents, I and II, with PS80-II constituting 12% of the total mass. Half-solid blue spheres represent phase-separated samples, while open symbols indicate single-phase samples. Light gray lines represent tie lines that could apply to selected samples, and the corresponding boundary of the two-phase range is solid gray. Alternative selections demonstrated in Figure S-8 gave rise to the short-dotted and dash-dotted, dark gray boundaries in A. Black squares in A denote sample compositions obtained by RP–UPLC–MS (open black squares: SPP, and solid: SRP), showing ECs on the PS80-I axis and NECs on the II axis. Small blue and black numbers in the plot denote the molarity of the sample corresponding to a certain data point.

For each sample under consideration, we selected plausible values for the concentrations of PS80-I and PS80-II in the SPP. It is important to note that both the extrapolation of *X*_PS80_ to φ_SRP_→0 and the appearance of an SRP at very low *c*_PS80_ implied a very low PS80 concentration (I + II) in the SPP, which is close to the buffer corner of the phase triangle. Then, the corresponding compositions of the SRP were obtained using the lever rule, constructing a tie line (light gray lines in Figure 7) crossing the blue sphere with its two ends (small light gray spheres) defining the bold dark gray phase boundary. The length of the left lever (the segment from the SPP to the blue sphere) represents the fractional volume of the SRP, φ_SRP_, relative to the entire tie line. The positions of the phase boundaries above the blue dashed line cannot be deduced from the data.

At 85 °C (Figure 7A), the corresponding phase diagrams (pseudobinary and pseudoternary) are closely aligned. The crucial point is that at least the high-concentration tie lines are close to parallel to the dashed blue line, i.e., our sample series approximately follows a tie line. Then, the pseudobinary behavior is to be expected even in a ternary system. Nevertheless, some independent redistribution of the NEC between SRP and SPP is indicated by the tie lines. Figure S-8 presents two more phase diagrams at 85 °C, each based on different but still reasonable assumptions regarding the SPP compositions. These alternative two-phase ranges are shown in Figure 7 as short-dotted and dash-dotted dark gray lines. While these alternative lines show some deviation from the bold line representing the primary model, the fundamental behavior of the system remains consistent across different representations.

Following the same procedure for the visual inspection data obtained at 75 °C leads to the phase diagram in Figure 7B. Here, we have the additional information that the tie lines must be closer to parallel to the base, *X*_PS80‑I_ axis to allow for the low φ_SRP_ = 30% at 40 mM to reach 100% below 80 mM (the tie line reaches far to the right from the blue sphere). Consequently, the blue line has a rather steep angle to the tie lines, indicating a strong redistribution of the components. It crosses the phase boundary and leaves the two-phase range just before reaching 80 mM PS80, close to a critical point. The proximity to this critical point explains the homogeneous opaque appearance of the single phase at 80 mM, while a clear dispersion is observed at 160 mM (see Figure 3A).

### 4.3 NEC Concentrations Measured in SRP are Comparable to Those Predicted for “Pseudocomponent II”

It is important to note that the individual concentration data of NEC in SPP and SRP, as obtained by RP–UPLC–MS, were not considered in constructing the phase diagrams. These data are depicted in Figure 7A by open black squares for SPP and solid black squares for SRP, labeled with *c*_PS80_ upon collecting the respective fractions. The fact that the open black squares are not on a straight line starting at the buffer corner (*X*_PS80‑I_ = *X*_PS80‑II_ = 0) illustrates that also at 85 °C, there is some redistribution of NEC between the phases as *c*_PS80_ changes. This is in line with the slope of the phase boundary.

Since the black squares for NECs and ECs obtained by UPLC–MS were established independently of the phase boundaries derived for I and II from visual inspection (plus setting *x*_II_: = 12%), a comparison between the two data sets allows to evaluate the hypothesis that NECs represent component II and ECs make up I. Inspection of Figure 7A shows significant though not drastic deviations, given experimental and conceptual uncertainties, between the tie line ends and the solid black squares corresponding to SRP samples collected at the same total concentration (consider black and blue labels for c_PS80_ in mM in Figure 7A). This observation suggests that while the fraction of all NECs may not precisely match pseudocomponent II, NEC components likely play a dominant role in pseudocomponent II and are significantly associated with the anomalous clouding behavior of PS80.

### 4.4 Role of Nonesterified Compounds as an Intrinsic Cosurfactant in PS80

The compounds comprised in PEG 400 and Renex S30 are part of the NEC fraction of PS80. Changing their relative content in the sample by adding free, extra PEG 400 or Renex S30 showed pronounced effects on the clouding behavior of PS80. PEG 400 reduced the concentration of PS80 in the SRP, i.e., it increased the water content in the SRP. The increasing slope of φ_SRP_(c_PS80_) is then explained by the recruitment of PEG 400 (along with water) into the SRP with the increasing total PS80 concentration. Overall, the lower water content of the SRP at higher Renex may be explained by its higher polarity, preferring the SPP and changing water partitioning in favor of the SPP. Another possible effect of Renex is to replace water in interaction within the SRP—a phenomenon similar to lyoprotective effects of sugars.

The partitioning of each of the intrinsic NEC compounds between SRP and SPP must be considered to change individually with the total PS80 concentrations. This explains the concentration-dependent changes in the phase compositions and the subsequent nonlinear progress of SRP formation. It is interesting to note that the two-phase range in Figure 7A is “lifting up” from the base axis. This means that the native content of NECs (12%) indicated by the blue dashed line favors a wide two-phase range up to a high PS80 concentration. A higher or lower NEC content would be represented by straight lines starting at the water corner (as the blue line) but having a higher or lower slope than the blue line; such lines are likely to reach the limit of the two-phase range at a lower total PS80 concentration. This might be related to our finding that some NECs promote and others oppose separation. In other words, clouding and generally dewetting phenomena are regulated by the subtle balance between ECs and NECs and, on top of this, the balance between different NEC species.

### 4.5 Pharmaceutical Perspective on Liquid–Liquid Phase Separation in Protein Solutions

Liquid–liquid phase separation (LLPS) in protein solutions has been extensively studied to understand the physical behavior of proteins in solution. LLPS occurs when a homogeneous protein solution is separated into two distinct liquid phases with varying protein concentrations. This phenomenon is influenced by both the intrinsic properties of the protein and the excipients within the formulation.^28–30^

Key protein-related factors such as concentration, structure, and isoelectric points play significant roles. Additionally, excipients and formulation conditions critically impact LLPS.^31^ Surfactants such as PS80 can alter surface tension and interfacial properties, thereby potentially affecting the phase behavior,^55^ and subsequently could lead to an LLPS in biopharmaceutical products.^31^ In the case of PS80, the factors driving the LLPS are further complicated by its heterogeneous composition.^49^ Therefore, it is essential to carefully manage PS80 characteristics to prevent its clouding, subsequently inhibit any possibly PS80-induced LLPS, and ensure the stability and efficacy of biopharmaceutical products containing PS80.

## 5 Conclusions

PS80 should not, even approximately, be viewed as a single-component surfactant. This is evident in the context of clouding, thermotropic liquid–liquid phase separation, where a pseudobinary phase diagram of PS80 and buffer fails to adhere to the lever rule, violating the basic thermodynamic laws for a two-component system. A much more consistent approach was obtained by considering PS80 to consist of two pseudocomponents giving rise to a ternary phase diagram with buffer. The data imply that nonesterified components (NECs) of PS80 show a concentration-dependent redistribution between the phases and hence contribute crucially to the second pseudocomponent.

Different NEC subfractions may have opposing effects on clouding. Specifically, the addition of low-molecular-weight poly­(ethylene glycol) (LMW–PEG) was found to allow for a higher water content in the SRP, thus reducing the PS80 concentration in the SRP. Increasing partitioning of PEG 400 into the SRP with increasing total PS80 enhances this effect, causing a nonlinearly increasing SRP fraction.

Conversely, adding ethoxylated sorbitan (Renex S30) progressively decreased the water content in the SRP with an increase in total PS80. This may result from the attraction of water to the SPP and/or the replacement (and release) of water in the SRP reminiscent of a lyoprotectant. The native content of NECs in PS80 HP seems optimal for a low water content in the SRP, leading to a wide two-phase range.

## Supplementary Material



## Notes

More raw data of visual inspection, both as photographs of the samples and φ_SRP_(*c*_PS80_) plots, including linear regression in the two-phase range; additional UPLC-MS elution profiles of *T*_CL_(*X*) for other surfactants taken from the literature (as discussed in the text); illustration of the different types of PS80 HP ingredients, and their fatty acid composition; and alternative pseudoternary phase diagrams to Figure 7 in the main text (PDF)
