# Supplementary material for: Anomalous Clouding Behavior of Polysorbate 80—Deciphering the Role of Nonesterified Components
Source: Mol Pharm. 2025 May 14;22(6):2917–26. doi: 10.1021/acs.molpharmaceut.4c01268 (PMC12135041; doi:10.1021/acs.molpharmaceut.4c01268)
Supplement: Supplementary file 1 [file mp4c01268_si_001.pdf]

Electronic Supporting Information for

Anomalous clouding behavior of Polysorbate 80 — deciphering the role of non-esterified components

Alaa Hassan[1,5*], Tim Diederichs[2], Patrick Garidel[2,3] & Heiko Heerklotz[1,3*]

[1] University of Freiburg, Institute of Pharmaceutical Sciences, Hermann-Herder-St. 9, 79104 Freiburg im Breisgau, Germany.
[2] PDB-TIP, Innovation Unit, Boehringer Ingelheim Pharma GmbH & Co. KG, Birkendorfer St. 65, 88397 Biberach an dem Riss, Germany.
[3] Martin Luther University Halle-Wittenberg | MLU · Institute of Chemistry
[4] University of Toronto, Lesile Dan Faculty of Pharmacy, 144 College St., Toronto, Canada
[5] Cairo University, Faculty of Pharmacy, Kasr El-Aini St., 11562 Cairo, Egypt
* Correspondence: heiko.heerklotz@pharmazie.uni-freiburg.de (H.H); alaa_hassan@pharma.cu.edu.eg (A.H)

Principal component-families in PS80 HP

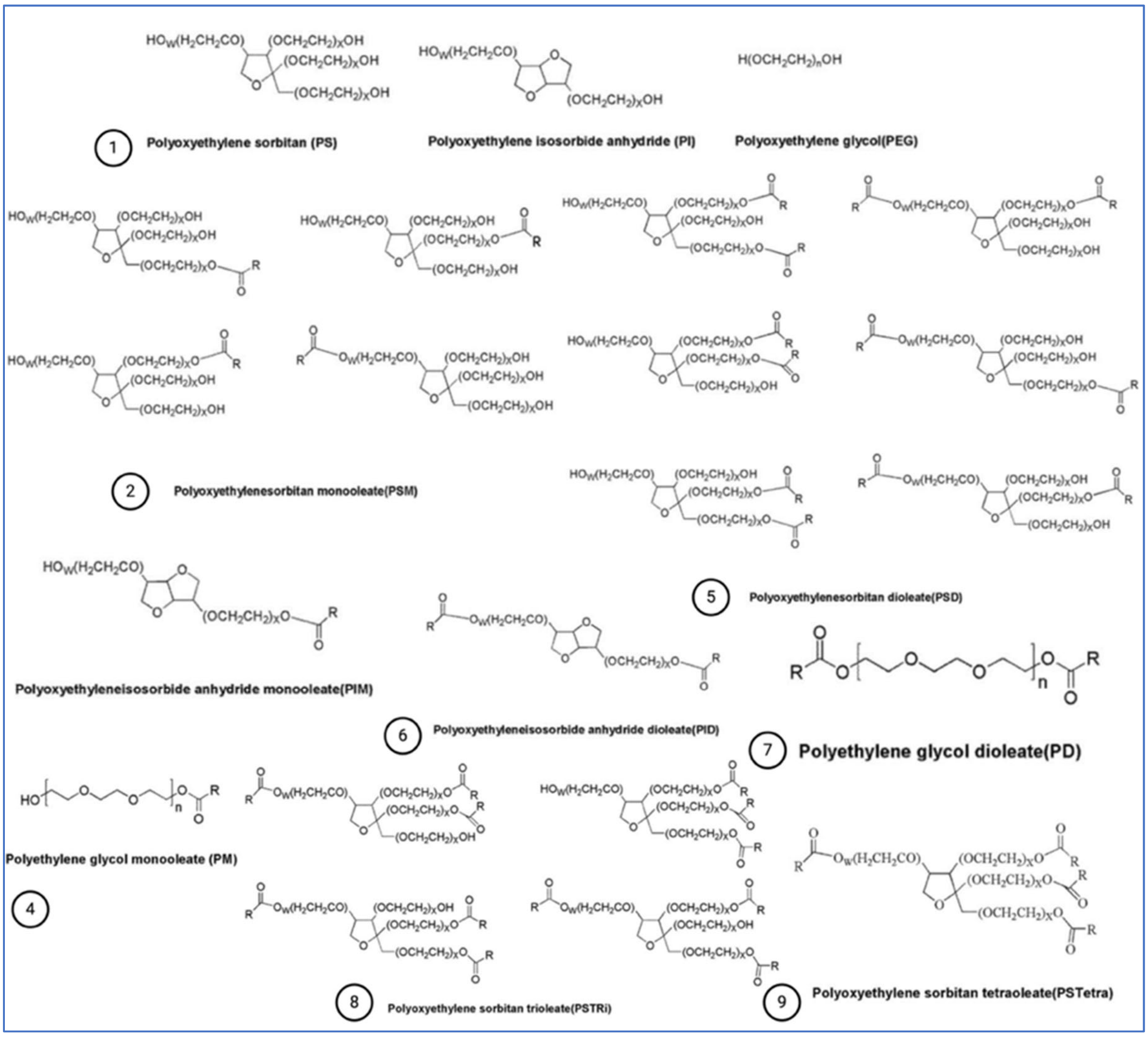

***Figure ESI-1: Distinct compositions of both injectable and non-injectable grades of PS80 HP from six manufacturers, including seven batches, and reported nine main species of compositions in PS80 HP** separated by reversed-phase HPLC and validated by UPLC-Q-TOF-MS (Adapted from [1] into a single composite using BioRender.com.*)

Fatty acid composition regarding PS80 HP.

Table ESI-1 *Overview of fatty acid composition of PS80 HP according to Ph. Eur.—European Pharmacopoeia 10.0 version [2], USP—United States Pharmacopeial Convention from 2017 [3], and JP—Japanese Pharmacopoeia, 18th edition [4].*

Fatty Acid	PS80 HP/ %
Myristic acid (C14:0)	≤ 5
Palmitic acid (C16:0)	≤ 16
Palmitoleic acid (C16:1)	≤ 8
Stearic acid (C18:0)	≤ 6
**Oleic acid (C18:1)**	**≤ 58**
Linolenic acid (C18:2)	≤ 18
Linolenic acid (C18:3)	≤ 4

Experimental: Principal approach and additional examples for visual inspection results:

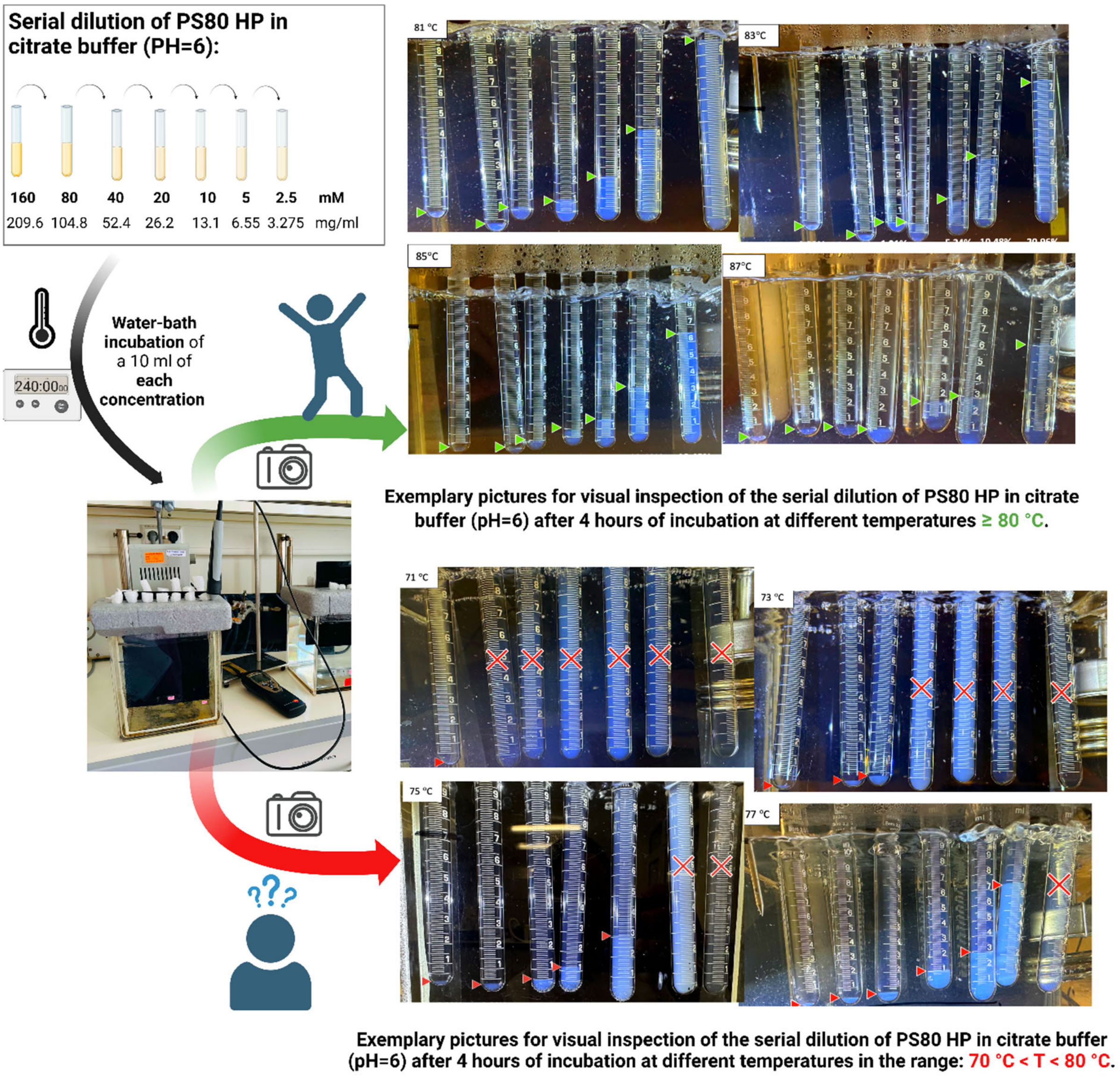

Figure ESI-2: Principal approach and documentary photographs *were taken to demonstrate the progression of the phase separation within the studied temperature range. It should be noted that only chosen photos are presented. (Illustration created using BioRender.com)*

Linear Regression Analysis analogous to Figures 2B, 3B in the main text

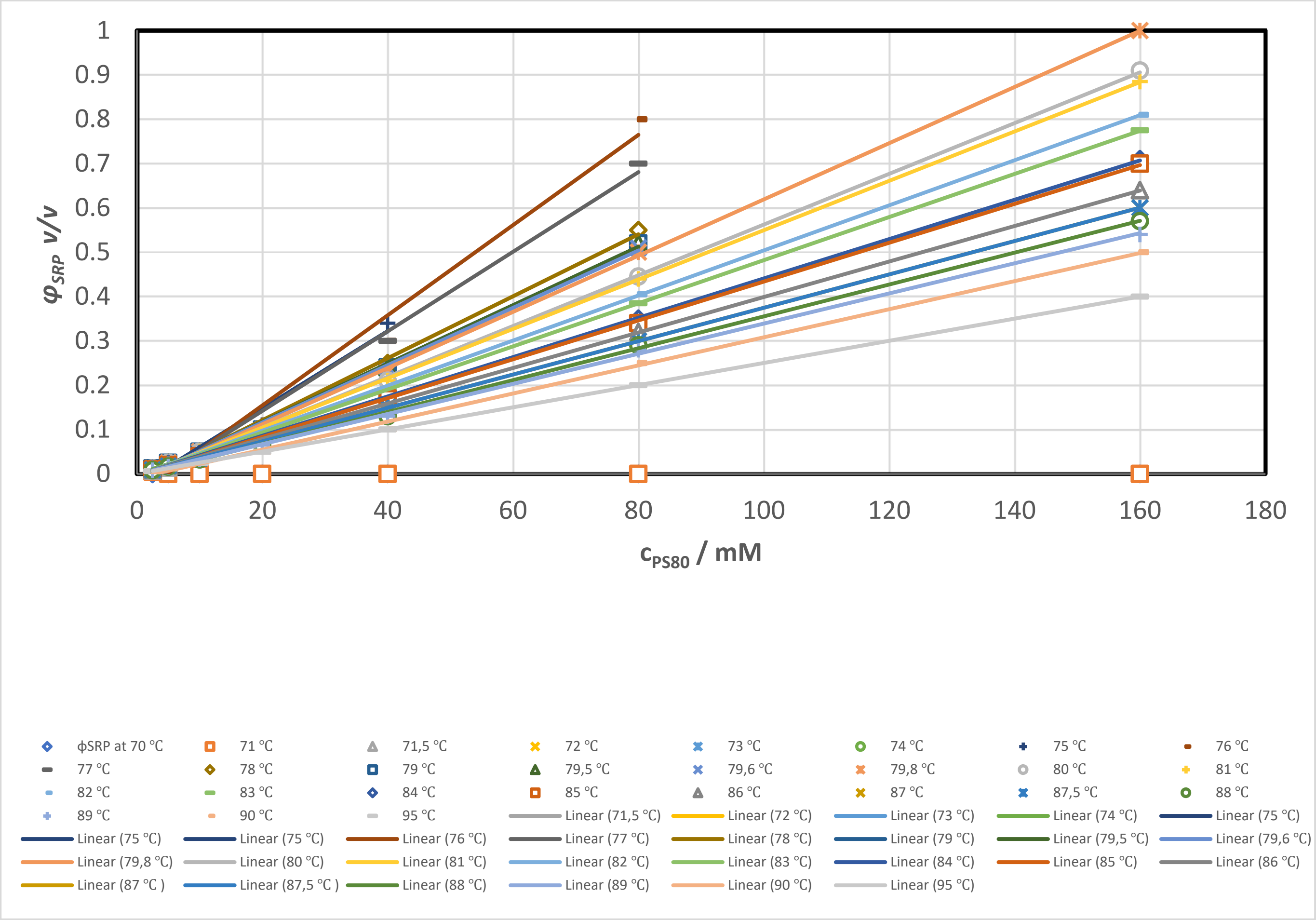

Figure ESI-3: Linear regression analysis *of the volume fraction of the surfactant-rich phase (φ_{SRP}) within the studied temperature range as a function of the surfactant concentration, analogous to Figure 2B and 3B in the main text for additional series.*

Attempt to construct pseudo-binary diagram, log abscissae

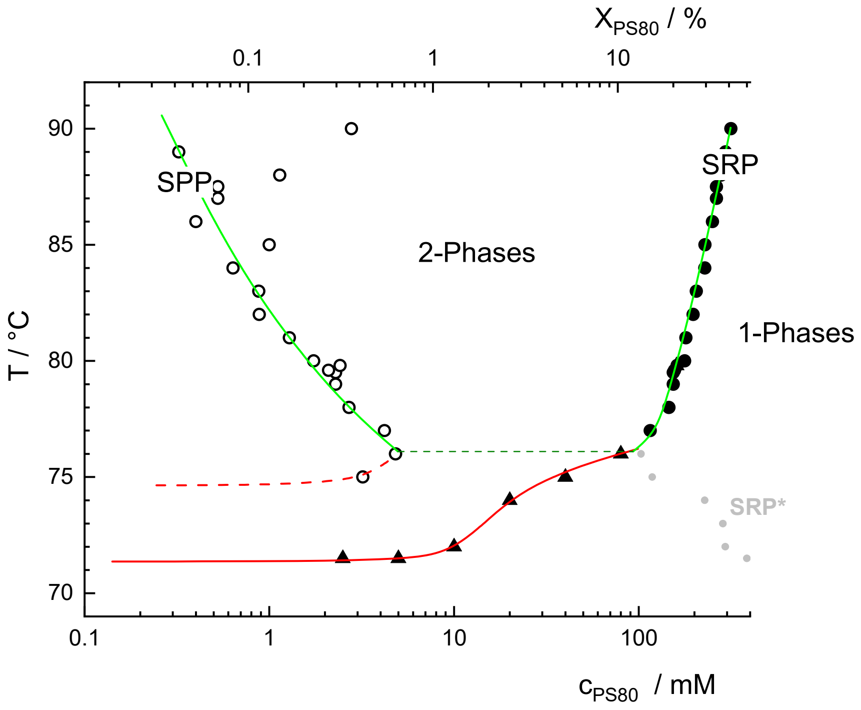

Figure ESI-4: *Reproduction of the* ***pseudo-binary phase diagram presented in" Figure 6" in the main text*** *– here with logarithmic abscissae for a better resolution of SPP results.*

Clouding "Phase diagrams" for nonionic surfactants in the literature

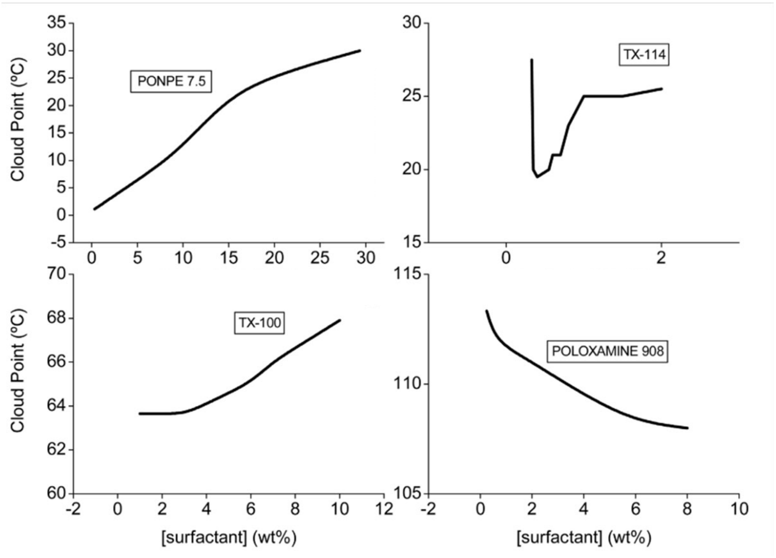

Figure ESI-5: *Variation of Cloud Point with surfactant concentration for four non-ionic surfactants (adapted from Figures of References* [5,6,7].

Experimental: Sample preparation for RP-UPLC-MS

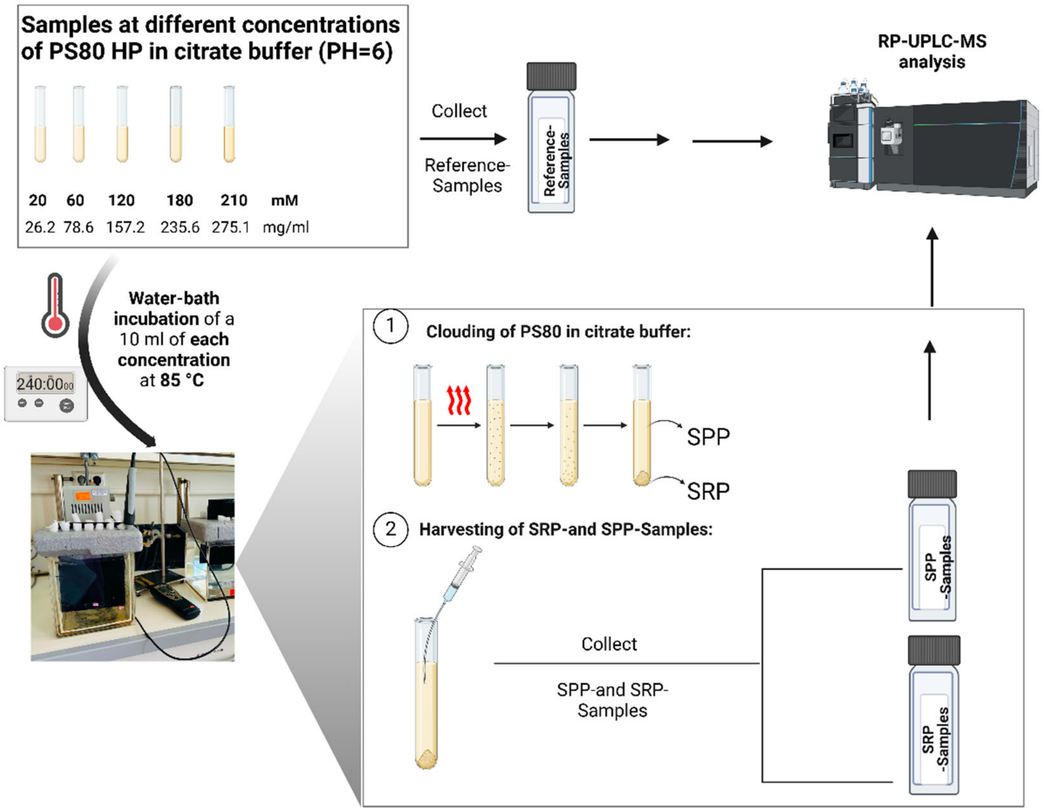

Figure ESI-6: Preparation of reference-, SPP- and SRP samples for analysis using RP-UPLC-MS: *Reference samples were collected from non-heated, non-separated solutions of different concentrations (20, 60, 120, 180, and 210 mM). Separated samples (surfactant-rich phase, SRP; surfactant-poor phase, SPP) were equilibrated at 85°C for 4 hours and harvested with extreme caution to avoid disturbing the separation between the two phases. All samples were analyzed using a reverse-phase ultra-performance liquid chromatography-mass spectrometry (RP-UPLC-MS) method, adapted from Lippold et al.* [8] *and Evers et al.* [9]*. (Illustration created using BioRender.com)*

Experimental: RP-UPLC-MS Analysis

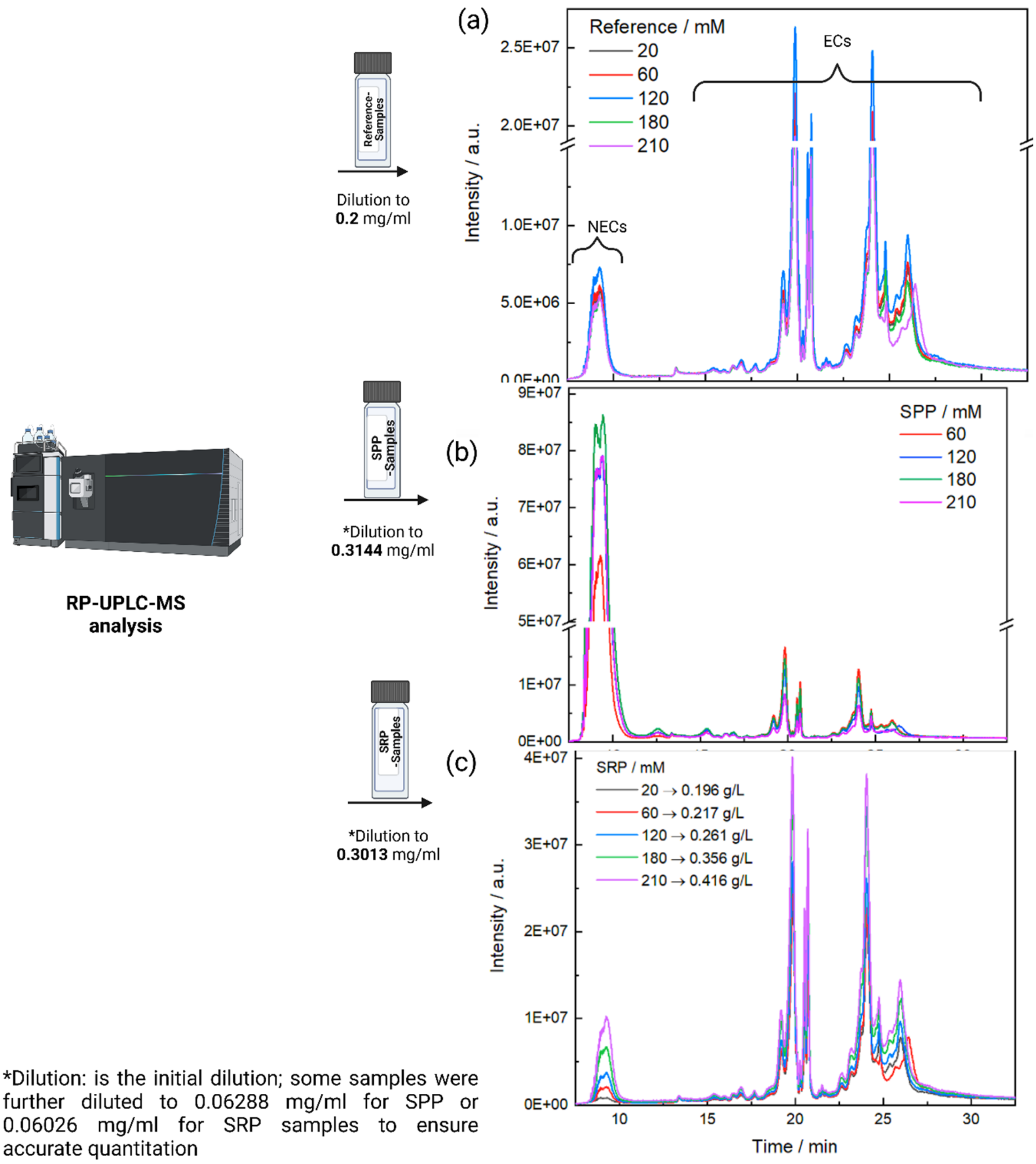

Figure ESI-7: RP-UPLC-MS analysis: A: *Actual chromatograms of reference samples (note: only the 120 mM sample is not a perfect fit); various concentrations of PS80 HP revealed consistent amounts of esterified components (ECs) and non-esterified components (NECs), accounting for 88% and 12% of the area under the curve (AUC), respectively,* ***B:*** *Actual chromatograms of SPP samples,* ***C:*** *Actual chromatograms of SRP samples, where the NECs distribute more favorably with increasing total concentrations of PS80 HP.*

Alternative examples for hypothetic ternary phase diagrams at 85°C (in line with visual inspection data)

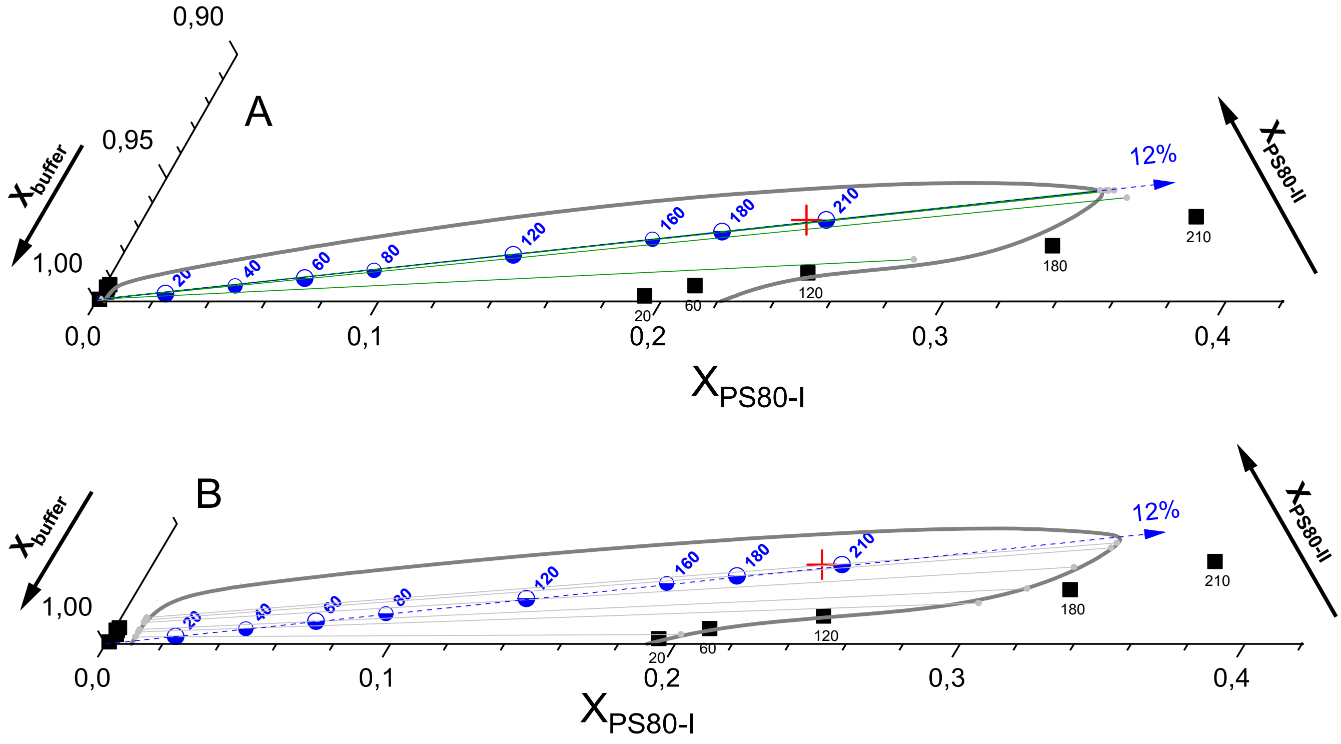

***Figure ESI-8: Alternative constructions of ternary phase diagrams** that would be in line with visual inspection results at 85°C in addition to that shown in Figure 7A (main text). The bold dark grey phase boundaries of A and B are included in Figure 7A as dotted and dash-dotted dark grey lines. Compared to Figure 7, **Panel A** was constructed for a lower content of PS80-II in the SPP, giving rise to steeper slopes of the tie lines, while **Panel B** is based on a higher content of PS80-I in the SPP; the shorter "left lever" at low c_{PS80} moves also the SPP-boundary to lower X_{PS80}.*

References:

(1) H. Sun; R. Yang; J. Wang; X. Yang; J. Tu; L. Xie; C. Li; Q. Lao; C. Sun. Component-Based Biocompatibility and Safety Evaluation of Polysorbate 80. *RSC Adv* **2017**, *7* (25), 15127–15138. https://doi.org/10.1039/c6ra27242h.

(2) Arzneibuchmonografie 10.0/0428 : Polysorbat 80 (Polysorbatum 80). In *European Medicines Agency (Eur. Arzneib. 10.0)*; 2020; pp 5369–5371.

(3) Polysorbate 80 Monograph. In *The United States Pharmacopeial Convention*; 2017.

(4) Official Monographs (from M to Z). In *Japanese Pharmacopoeia 18th Edition*; 2021.

(5) Hinze, W. L.; Pramauro, E.; Poole, C. F. A Critical Review of Surfactant-Mediated Phase Separations (Cloud-Point Extractions): Theory and Applications. *Crit Rev Anal Chem* **1993**, *24* (2), 133–177. https://doi.org/10.1080/10408349308048821.

(6) Naqvi, A. Z.; Khatoon, S.; Kabir-Ud-Din. Phase Separation Phenomenon in Non-Ionic Surfactant TX-114 Micellar Solutions: Effect of Added Surfactants and Polymers. *J Solution Chem* **2011**, *40* (4), 643–655. https://doi.org/10.1007/s10953-011-9671-9.

(7) George C. Na; Barbara O. Yuan; H. Jack Stevens Jr.; Brian S. Weekley; Natarajan Rajagopalan. Cloud Point of Nonionic Surfactants: Modulation with Pharmaceutical Excipients. *Pharmaceutical Research* **1999**, *16* (4), 562–568.

(8) Lippold, S.; Koshari, S. H. S.; Kopf, R.; Schuller, R.; Buckel, T.; Zarraga, I. E.; Koehn, H. Impact of Mono- and Poly-Ester Fractions on Polysorbate Quantitation Using Mixed-Mode HPLC-CAD/ELSD and the Fluorescence Micelle Assay. *J Pharm Biomed Anal* **2017**, *132*, 24–34. https://doi.org/10.1016/j.jpba.2016.09.033.

(9) Evers, D. H.; Schultz-Fademrecht, T.; Garidel, P.; Buske, J. Development and Validation of a Selective Marker-Based Quantification of Polysorbate 20 in Biopharmaceutical Formulations Using UPLC QDa Detection. *J Chromatogr B Analyt Technol Biomed Life Sci* **2020**, *1157*. https://doi.org/10.1016/j.jchromb.2020.122287.
